# Pirfenidone Accelerates Wound Healing in Chronic Diabetic Foot Ulcers: A Randomized, Double-Blind Controlled Trial

**DOI:** 10.1155/2017/3159798

**Published:** 2017-12-31

**Authors:** Luz E. Gasca-Lozano, Silvia Lucano-Landeros, Héctor Ruiz-Mercado, Adriana Salazar-Montes, Ana Sandoval-Rodríguez, Jesus Garcia-Bañuelos, Arturo Santos-Garcia, Judith R. Davila-Rodriguez, José Navarro-Partida, Hiram Bojórquez-Sepúlveda, Juan Castañeda-Gomez, José Domínguez-Rosales, Myriam A. Ruiz-Arcos, María Guadalupe Sánchez-Parada, Juan Armendariz-Borunda

**Affiliations:** ^1^Institute for Molecular Biology and Gene Therapy, CUCS, University of Guadalajara, Guadalajara, JAL, Mexico; ^2^Regional Hospital Dr. Valentín Gómez Farías ISSSTE, Guadalajara, JAL, Mexico; ^3^Tecnologico de Monterrey, Campus Guadalajara, Jalisco, Mexico; ^4^Hospital Civil de Guadalajara, Guadalajara, JAL, Mexico; ^5^Institute of Chronic-Degenerative Diseases, CUCS, University of Guadalajara, Guadalajara, JAL, Mexico

## Abstract

**Background:**

Diabetic foot ulcers are one disabling complication of diabetes mellitus. Pirfenidone (PFD) is a potent modulator of extracellular matrix. Modified diallyl disulfide oxide (M-DDO) is an antimicrobial and antiseptic agent.

**Aim:**

To evaluate efficacy of topical PFD + M-DDO in a randomized, double-blind trial versus ketanserin in the treatment of noninfected chronic DFU.

**Methods:**

Patients received PFD + M-DDO or ketanserin for 6 months. Relative ulcer volume (RUV) was measured every month; biopsies were taken at baseline and months 1 and 2 for histopathology and gene expression analysis for COL-1*α*, COL-4, KGF, VEGF, ACTA2 (*α*-SMA), elastin, fibronectin, TGF-*β*1, TGF-*β*3, HIF-1*α*, and HIF-1*β*.

**Results:**

Reduction of median RUV in the PFD + M-DDO group was 62%, 89.8%, and 99.7% at months 1–3 and 100% from months 4 to 6. Ketanserin reduced RUV in 38.4%, 56%, 60.8%, 94%, 94.8%, and 100% from the first to the sixth month, respectively. Healing score improved 4.5 points with PFD + M-DDO and 1.5 points with ketanserin compared to basal value. Histology analysis revealed few inflammatory cells and organized/ordered collagen fiber bundles in PFD + M-DDO. Expression of most genes was increased with PFD + M-DDO; 43.8% of ulcers were resolved using PFD + M-DDO and 23.5% with ketanserin.

**Conclusion:**

PFD + M-DDO was more effective than ketanserin in RUV reduction.

## 1. Introduction

Around 415 million of people in the world are living with diabetes mellitus type 2, representing 8.3% of the world population as of 2015 [1]. Diabetic foot ulcers (DFU) are one of the main disabling complications of this disease, and it is estimated that up to 13% of people residing in North America with type 2 diabetes will develop a foot ulcer during the course of their lives [2]. The above information represents a significant low limb amputation risk factor [3], since it is known that 85% of amputations due to diabetes are preceded by ulcers [4].

Evolution of patients who have undergone a major amputation is negative; as much as 44% of these patients die during the first year and it is estimated that 77% of them will have passed away within 5 years [5].

The underlying pathology of diabetic foot ulcer is neuropathy, with or without the presence of ischemia, peripheral vascular disease, and infection associated with failure to heal and possible amputation. All these complications are associated with chronic sensor-motor neuropathy and vascular disease [6], transforming growth factor beta 1 (TGF-*β*1) and hypoxia-inducible factor 1-alpha (HIF-1*α*) downregulation [7–9], high levels of tumor necrosis factor alpha (TNF-*α*) and oxidative stress [10], delayed expression of the keratinocyte growth factor (KGF) [11], and impaired immune function.

It is known that the TGF-*β* family plays a crucial role in wound healing. Under normal conditions, when the skin suffers an injury, different mechanisms are set in place. These processes, whose sequence overlaps over time, are executed by different cells, both epithelial and blood cells, orchestrating the repair of damaged tissue [12].

TGF-*β*1 promotes differentiation of fibroblasts to myofibroblasts, migration, and cell proliferation, stimulates keratinocytes to produce laminin among other constituents of the normal basement membrane, and stimulates myofibroblasts to contract [13].

However, in diabetic patients with hyperglycemia, there is a decrease in the expression of TGF-*β*1. This decrease delays KGF expression [11], differentiation of fibroblasts to myofibroblasts, and the production of a-SMA [14]. In addition, the intimate relationship of TGF-*β*1 in monocyte chemotaxis, in inflammatory reaction, and in cellular response could contribute to diminished immune function and the poor reaction of leukocytes to pathogenic organisms [15]. This, together with elevated levels of tumor necrosis factor alpha (TNF-*α*) [10] and increased oxidative stress [16], causes delayed epithelialization and exposes patients to possible infections.

Janka-Zires et al. recently conducted a randomized crossover clinical trial to evaluate the effect of pirfenidone and conventional treatment and found that when patients were switched from conventional care to pirfenidone treatment, this significantly promoted wound healing in uninfected DFU, with a percentage of 52.4% of wounds totally healed in eight weeks [17].

Pirfenidone (PFD) is an antifibrogenic molecule used for the treatment of idiopathic pulmonary fibrosis [18]. PDF inhibits the proliferation of myofibroblasts and reduces the expression of *α*-SMA in the presence or absence of TGF-*β*1. In addition to considerably decreasing the formation of COL-1*α* [19], it is also an antioxidant and anti-inflammatory agent successfully used in pathologies associated with inflammation and oxidative stress. PFD reduces secreted and cell-associated TNF-*α* levels [20] and oxidative stress [21, 22].

In Mexico, we normally follow the guidelines of the International Consensus on the Diabetic Foot [23]; however, we concomitantly use wound-healing enhancers. The most commonly used is ketanserin (KTS) which has been approved for wound treatment by The Federal Commission for the Protection against Sanitary Risk (COFEPRIS) under registry number 259M90 SSA [24].

KTS (3-[2-[4-(4-fluorobenzoyl)piperidin-1-yl]ethyl]-1H-quinazoline-2,4-dione) is a quinazoline derivative, a serotonin antagonist of 5-HTR2, with no agonistic properties [25].

KTS has been used in several clinical trials for treating diabetic foot ulcers. Janssen et al. used 2% KTS to improve wound healing in different kinds of patients, including 6 patients with diabetes. They reported that 36% of ulcers healed in the KTS group as opposed to 15% in the placebo group at 8 weeks [26]. In Sweden, Apelqvist et al. evaluated KTS in diabetic foot ulcers with severe peripheral vascular disease. They found that 56% of patients with a toe pressure below 30 mmHg improved their ulcers in contrast with 11% in the placebo group [27].

More recently in Mexico, Martínez-de Jesús et al. tested 2% topical KTS in diabetic foot ulcers. They reported that an 87% reduction of ulcer area at 12 weeks contrasted with 63% in the placebo group [28]. Quatresooz et al. performed a double-blind intraindividual comparative study to revisit the effect of 2% topical KTS in patients with diabetes and venous insufficiency in Belgium. They reported a 94% reduction of relative wound area against 32% in the placebo group [29]. Pursuant to previous findings, we decided to assess the effectiveness of PFD + M-DDO in treating noninfected chronic DFU in a randomized, controlled double-blind trial versus KTS. We also determined the effect on the expression of cardinal genes related to the wound healing process.

## 2. Materials and Methods

### 2.1. Methodological Design End Ethics

The study was designed as a single-center, randomized, double-blind, active-controlled trial. Patient enrollment took place at the Dr. Valentín Gómez Farías Regional Hospital pertaining to the ISSSTE system in Guadalajara, Mexico, between 2014 and 2015. The clinical trial was approved by The Ethical Review Board of the Dr. Valentín Gómez Farías Regional Hospital, performed in accordance with the Ethical Principles of the Declaration of Helsinki, and took into account the Good Clinical Practice guidelines. The trial was registered at ClinicalTrial.gov under registration ID: NCT02632877 before participant enrollment. All the participants provided their written informed consent.

### 2.2. Participants

Patients with a previous diagnosis of DM2 according to the ADA criteria were enrolled. All of them were under pharmacological treatment for glycemic control, had at least one-foot ulcer classified as A-I following the University of Texas Diabetic Wound Classification (UTDWC), and had at least a 2-month duration. Patients were randomly assigned to receive one of two interventions.

Inclusion criteria are as follows: men and women with diabetes mellitus type two, over 18 years of age, and with a DFU grade A-I following the University of Texas Diabetic Wound Classification > 1cm^2^ persisting for a minimum of 2 months.

Exclusion criteria are as follows: patients who required either direct (graft) or indirect revascularization procedures during the study, major large-vessel and peripheral arterial disease, grade III insufficiency of the deep venous system assessed by means of the ankle-arm index of 0.9 to 0.7, and autoimmune disease; pregnancy or breast-feeding; inability to attend the monthly evaluations; and patients who, within a period of less than seven days, had applied any topical application to the ulcer, whether pharmacological or not, apart from the water and soap used in the cures.

**Figure 1:**
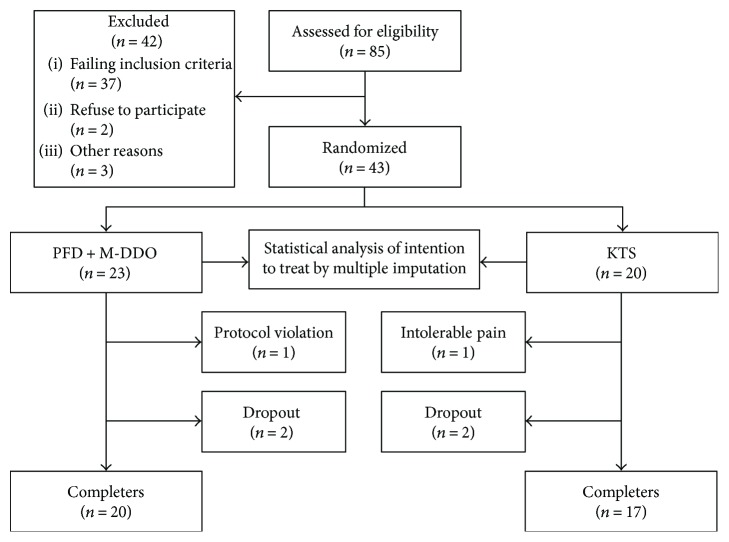
Enrollment.

Elimination criteria are as follows: participants with <80% scheduled medical appointments, absence from more than 20% of the visits, with serious side effects or allergic reactions, severe to moderate pain, erythema, edema, and/or necrosis were removed from the study, though all of them were considered for the final statistical analysis (Figure 1). Compliance to the treatment was assessed by the number of applications that were recorded by the patient in an attendance logbook and by the retrieved medication container.

### 2.3. Randomization and Hidden Allocation

Eligible participants were enrolled and randomly assigned to experimental and active control groups using a random number table. Patients, medical doctors ascribed to the hospital who were in charge of clinical care, the statistical evaluator, and histopathological evaluator were blinded. Concealment was opened at the end of the study.

### 2.4. Interventions and Dosage

Kitocell-Q®, a drug in the form of a gel that combines PFD 8% and M-DDO 0.016%, was obtained from Cell Pharma, SA de CV (México City, México) while Sufrexal® (2% KTS) was acquired from Janssen Pharmaceuticals (New Jersey, USA). Additionally, patients in both groups received integral and conventional care according to the International Consensus on the Diabetic Foot [23]. Conventional care consisted of relieving pressure, metabolic control, and local wound care with frequent wound debridement and absorbent, nonadhesive, nonocclusive dressings by a multidisciplinary team. Patients were applied either KTS gel 3 times per day (t.p.d.) or PFD + M-DDO 2 t.p.d. topically in the ulcer area over the entire extension of the ulcer in a thin layer for six months according to their dosage. The follow-up study lasted six months. Three months were given to afford the ulcer the opportunity to close and three more months to verify that the ulcer did not reopen, according to the recommendations of the FDA [30].

### 2.5. Outcomes and Data Collection

Relative ulcer volume (RUV) was assigned as a primary outcome. Immediately before the intervention, a physician measured patients' diabetic foot ulcer (DFU) using a flexible sterile ruler. Criteria to measure the size of the wound consisted of assessing the longest, widest, and deepest sides. A photograph was taken later and the assigned sides of DFU were marked on it and kept in the file to serve as a guide to the physician for the following evaluation. Measurements were performed at baseline, 4, 8 12, 16, 20, and 24 weeks, and a photographic record was taken at the same time as a guideline.

RUV was obtained by multiplying the measure of the longest, widest, and deepest extension of the ulcer, according to the formula:

(1) $$\begin{array}{c} RUV=\left(L\right)\left(W\right)\left(D\right), \end{array}$$

where *L* = the longest extension of DFU in centimeters, *W* = the widest extension of DFU in centimeters, and *D* = the deepest extension of DFU in centimeters.

In order to evaluate the reduction percentage of DFU, baseline RUV for each patient was normalized to 100%. Reduction of median RUV was assessed for each month according to the formula:

(2) $$\begin{array}{c} reduction\text{ of median }\%=\frac{\left(\text{median of }{RUV}_{n}\right)\left(100\right)}{\text{median of }{RUV}_{0}}, \end{array}$$

where RUV_*n*_ = median of relative ulcer volumes at month 1, 2, 3, 4, 5, or 6 and RUV_0_ = median of relative ulcer volumes at baseline.

Secondary outcomes were wound healing, wound healing histopathological score, molecular assessment of genes involved, and safety and tolerability. Biopsies of approximately 125 mm^3^ were taken from the middle of ulcers at the beginning of interventions and at 1 and 2 months of treatment to accomplish the secondary outcomes. We did not take biopsies beyond three months in order not to reopen healed ulcers and not put patients at risk. Wound healing was defined in this trial as the percentage of healed wounds at the end of the study (24 weeks).

The wound healing histopathological score was assessed to evaluate the healing process of DFU. It was assessed according to a previous report [31] in the histological sections of biopsies. These were stained with hematoxylin-eosin and Masson's trichrome staining and evaluated by a histopathologist before concealment was opened.

A high score represents an improvement of healing and a low score a delay thereof. Parameters assessed in wound healing histopathologic score were as follows: amount of granulation tissue (profound—1, moderate—2, scanty—3, and absent—4), inflammatory infiltrate (plenty—1, moderate—2, and a few—3), collagen fiber orientation (vertical—1, mixed—2, and horizontal—3), the collagen pattern (reticular—1, mixed—2, and fascicle—3), the amount of early collagen (profound—1, moderate—2, minimal—3, and absent—4), and the amount of mature collagen (profound—1, moderate—2, and minimal—3). The total healing score was calculated by adding the scores of individual criteria, when the score was directly proportional to wound healing.

Molecular assessment was performed to elucidate the effect of treatments on the molecules involved in wound healing over time. Molecular assessment consisted of the evaluation of gene expression for type IV collagen (Col-4), type I collagen alpha 1 (Col-*α*1), transforming growth factor beta 1 (TGF-*β*1), transforming growth factor beta 3 (TGF-*β*3), vascular endothelial growth factor (VEGF), elastin, fibronectin, alpha-smooth muscle actin (*α*-SMA), keratinocyte growth factor (KGF), hypoxia-inducible factor 1-alpha (HIF-1*α*), and hypoxia-inducible factor 1-beta (HIF-*β*) by real-time quantitative reverse transcription (qRT-PCR). Gene expression was assessed based on biopsies and was compared with baseline, as explained below.

In this trial, a treatment was considered safe and tolerable if serious adverse effects were not observed during the study. Serious adverse events are assumed in this study as events that put the patient's life at risk, according to FDA 21312.32 Code of Federal Regulations.

Irritation, photosensitivity, intolerable pain, and intolerable burning were considered expected adverse events and grounds for suspending treatment. Any other adverse event was considered an unexpected event and was considered as grounds for suspending treatment according to the criteria of the attending physician.

Blood sampling and biochemical analyses were performed before interventions and at the end of the study. All blood sampling and biochemical analyses were performed at the hospital's clinical analysis laboratories and data were obtained from the records. Demographics were recorded at enrollment as were clinical data throughout treatment.

### 2.6. Sample Processing

The ulcer area was washed with aseptic solution (Accua Aseptic® solution, Cell Pharma, México) and a biopsy (100–150 mm^3^) was taken using a scalpel blade from the middle of the ulcer. Biopsies were taken at baseline, 1st month (4 weeks), and 2nd month (8 weeks) if the ulcer had not healed. Photographs were taken at all times and relative ulcer volume was measured monthly until complete healing. Relative ulcer volume (RUV) was calculated by measuring the longest, widest, and deepest ulcer side with a sterile flexible graduated ruler, as described previously. Blood samples were obtained at the baseline and at the end of the study to measure biochemical tests and to evaluate any side effects of KTS or PFD + M-DDO in biochemical parameters.

### 2.7. Alpha-SMA Immunohistochemistry

Biopsies (100–150 mm^3^) were taken using a scalpel blade from the middle of the ulcer at baseline, 1st month, and 2nd month after treatment with either PFD or ketanserin. Anti-human smooth muscle actin (*α*-SMA) antibody was obtained from Boehringer (Mannheim, Germany), histological-processed wound sections were deparaffinized, and endogenous activity of peroxidase was quenched with a solution 0.03% H2O2 in methanol. The tissue was incubated with a 1/100 dilution of a monoclonal mouse anti-human *α*-SMA antibody. Anti-mouse peroxidase-labeled secondary antibody was revealed with diaminobenzidine, and the tissue was counterstained with Harris's hematoxylin. Twenty random fields were evaluated for quantification at 200x magnification. The immunohistochemical positive area was measured with an automated analyzer (Image Pro 6.3, Qwin, Leica). Data are expressed as percentage of the *α*-SMA stained area.

### 2.8. RT-PCR

Total RNA was isolated from a portion of the biopsy using Trizol® reagent (Invitrogen®, Carlsbad, CA, USA) according to the Chomczynsky and Sacchi modified technique [32]. 300 ng of total RNA was employed for reverse transcription using MMLV reverse transcriptase (Invitrogen). Then, 2 *μ*L of cDNA was subjected to real-time PCR using specific TaqMan primers and probes designed to align in Col-4, Col-*α*1, TGF-*β*1, TGF-*β*3, VEGF, elastin, fibronectin, *α*-SMA, KGF, HIF-1*α*, and HIF-1*β* (Applied Biosystems Hammonton, NJ, USA). Gene expression was normalized against the housekeeping gene 18S. Relative quantification was achieved by using the 2^−∆∆CT^ method [33]. Gene expression levels are reported as relative expression units (REU).

### 2.9. Histological Processing

A portion of the biopsy was fixed at 4% paraformaldehyde, embedded in paraffin, and cut into 5 *μ*m thick tissue sections. Tissues were stained with hematoxylin-eosin and Masson's trichrome staining to determinate inflammation and extracellular matrix deposit. The healing score of each ulcer was calculated using a methodology recently reported [31] as described above.

### 2.10. Statistics

An intention to treat analysis (ITT) was performed using a multiple imputation method for missing data. Nonparametric tests (Mann–Whitney *U* test and Wilcoxon signed-rank), parametric test (Student's *t*-test), and descriptive statistics of quantitative and qualitative variables were used but due to the kurtosis of the data, which did not behave parametrically, it was not possible to analyze them using chi-square (*χ*2). A 5% probability was considered to show that differences were not due to chance (*p* < 0.05). SPSSv20 software was used to analyze the data.

## 3. Results

### 3.1. Enrollment

Eighty-five patients were screened from medical consultation at the Angiology Department to evaluate their inclusion in the protocol. Forty-three subjects were enrolled in the study (23 in PFD + M-DDO group and 20 in KTS group), 37 were excluded for failing the inclusion criteria, 2 refused to participate, and 3 for other reasons. Three patients were eliminated from the PFD + M-DDO group, 1 for protocol violation and 2 dropped out. In the KTS group, 3 patients were eliminated, 1 due to intolerable pain and 2 for missing follow-up. At the end of the study, 20 patients in the PFD + M-DDO group and 17 in the KTS group had concluded the trial (Figure 1).

**Figure 2:**
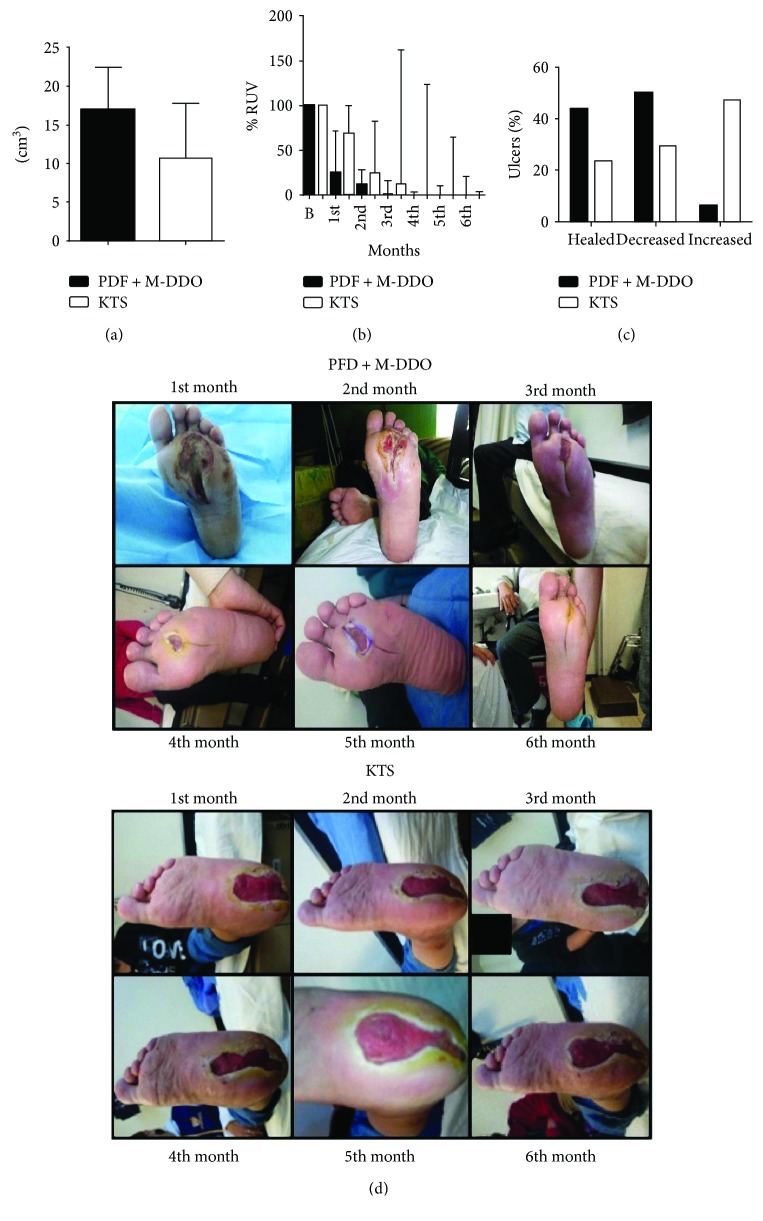
Ulcer parameters and evolution of ulcers throughout the treatment. (a) Relative volume of ulcer at baseline shows no difference between groups (*p* = 0.257). (b) Change of relative volume of ulcer over time. Differences are significant in the first three months of treatment (*p* = 0.036, *p* = 0.031, and *p* = 0.033; months 1–3). (c) Status of ulcers at the end of the six-month intervention. Data are expressed as mean ± SD. Representative photographs of a patient from each treatment group are shown, showing that PDF + M-DDO induces faster wound healing when compared to KTS.

Both groups were comparable at the beginning of the study. All subjects presented similar age, sex, ulcer classification according to UTWCS, RUV, and biochemical parameters. Demographic and clinical characteristics of these patients are shown in Table 1. Biochemical parameters were not modified at the end of any treatment (data not shown). In addition, no significant differences (*p* = 0.257) were found in RUV at baseline between the groups (Figure 2(a)). The Michigan Neuropathy Screening with altered cut point [34] was used to find out whether patients had neuropathy as mentioned above. Clinical data suggested that all patients have undergone some degree of neuropathy.

Five patients in the PFD + M-DDO group required debridement at some stage of this clinical trial, while 8 patients in the KTS group required it. Three patients in the KTS group had infections in the DFU in the course of the study and were treated with systemic antibiotics.

## 4. Primary Outcome

### 4.1. Relative Ulcer Volume (RUV)

It is evident that the major decrease in RUV (median + IQR) takes place earlier in the wound healing process induced by the combined drugs of PFD + M-DDO (Figure 2(b)). Significant differences between treatments were observed during the first three months (*p* = 0.036, *p* = 0.031, and *p* = 0.033, resp.).

Conclusions can be drawn from Figure 2(b) that is evident that the therapeutic effect induced by KTS in wound closure is delayed when compared to PFD + M-DDO. It should be taken into account that necessary debridement caused an increase in some months of RUV.

Significant reduction of RUV was found in the PFD + M-DDO group of patients as early as the first month and up to the sixth month when compared with baseline RUV. Improvement of median RUV in patients included in PFD + M-DDO group was 62% in the first month (*p* = 0.001), 89.8% in the second month (*p* < 0.001), and 99.7% in the third month (*p* < 0.001), respectively. From the fourth up to the sixth month, a 100% RUV was found (*p* < 0.001).

As for the KTS group, reduction of median RUV was 38.4% (*p* = 0.010), 56% (*p* = 0.003), 60.8% (*p* = 0.049), 94% (*p* = 0.067), 94.8% (*p* = 008), and 100% (*p* < 0.001) from the first to the sixth month, respectively (Figure 2(b)). The evolution of these injuries was evident at each physical examination and reflected in the representative patients shown in Figure 2(d).

## 5. Secondary Outcomes

### 5.1. Wound Healing

Interestingly enough, at the end of the study (six months), 43.8% of wounds had healed completely in the PFD + M-DDO group, 50% of them presented a decrease and 6.2% increased in size due to needed debridement. On the other hand, only 23.5% of ulcers were found entirely healed in KTS group, 29.4% decreased and 47.1% worsen compared to the RUV baseline (Figure 2(c)). Noteworthy, and as the topical treatment went on, an important number of KTS patients showed slower wound healing associated with complications such as newly incoming clinical infections (15%) with subsequent debridement and wound opening. No patient in the PFD group had infections in the course of the trial, and no healed ulcer presented reopening in either group during the follow-up period.

### 5.2. Wound Healing Histopathological Score

Histopathological analyses were conducted on tissue biopsies obtained as indicated in the section of Materials and Methods, in order to determine whether differences could be found in wound evolution among patients. Biopsies were read in a blind fashion by a certified pathologist and validated according to Gupta and Kumar [31].

**Figure 3:**
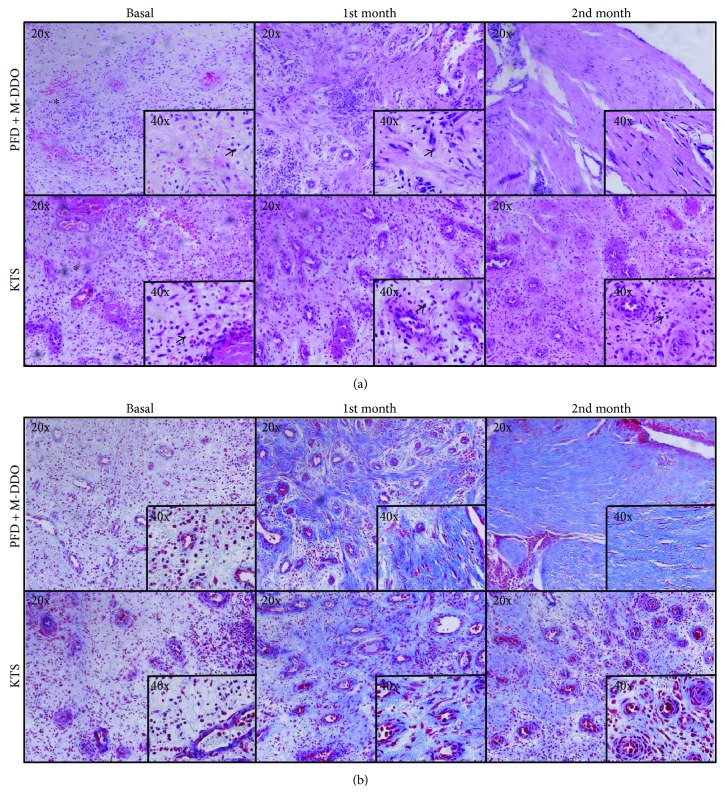
Wound healing histopathological analysis. Representative microscope photographs of ulcers in each treatment group are shown. (a) Hematoxylin-eosin staining, asterisks and arrows indicate inflammatory cells. (b) Masson's trichrome staining shows that PDF + M-DDO induced an abundant synthesis of ECM, and its accumulation in healed ulcers is evident when compared to KTS. In addition, the ordered deposition of collagens is clear. Photos were taken with 40x and 20x objective.

Patients in both groups showed a similar wound healing histopathological score at basal time (before intervention) in both groups: PFD + M-DDO 11 (9.5–15) [mean (IQR)] and KTS 11 (10–13) [mean (IQR)], (*p* = 0.621) (Figure 3(a), Table 2). The tissues had edema, hemorrhage, a great amount of inflammatory cell infiltrate with neutrophil and monocyte presence, fibroblasts, congested vessels, and scarce extracellular matrix accumulation at baseline (Figure 3(b)).

At the first month of treatment, PFD + M-DDO had an improvement of one point in the healing score 12 (10–15) (*p* = 0.591) and KTS did not show any change 11 (10–12.5) (*p* = 0.648) and no significant difference was observed (*p* = 0.273) (Table 2). Nonetheless, PFD + M-DDO reduced inflammatory infiltrate and increased collagen deposition more than KTS. It is clear that there is a substantial decrease in the number of inflammatory cells (Figure 3(a)) and abundant mature collagen, which looks organized and composed of aligned fiber bundles in PFD + M-DDO patients (Figure 3(b)). Thus, granulation tissue recruitment took place faster than in patients treated with KTS.

In the second month, the PFD + M-DDO group had a significant increment of 4.5 points [15.5 (14.3–16)] (*p* = 0.023) in the healing score (Table 2). Remarkably, inflammatory cell infiltrate was almost absent (Figure 3(a)). On the other hand, patients in the KTS group still had inflammatory cell infiltrates (Figure 3(a)), and the extracellular matrix stained by Masson's trichrome staining looked less abundant and clearly disorganized (Figure 3(b)) as compared with their PFD + M-DDO patient counterparts. The histopathological healing score revealed only a 1.5 point improvement [12.5 (11–16.3)] (*p* = 0.516) over the first month after KTS treatment, and significant differences were found between treatments (*p* = 0.050) (Table 2).

### 5.3. Molecular Assessments

We then searched for the molecular mechanisms involved in this accelerated wound healing in diabetic foot ulcers induced by PFD + M-DDO.  Table 3 shows an extensive analysis of gene expression of a number of key molecules involved in the entire wound healing process.

The results shown in Table 3 confirm and extend our previous observations shown in Figures 2 and 3. They clearly correlate with the expression of genes in extracellular matrix synthesis and collagen maturation, fibroblast and keratinocyte cell recruitment and proliferation, induction of a tissue regeneration process, and induction of angiogenesis needed for the formation of new blood vessels.

A significant, in some cases dramatic, increase in the expression of these genes at the first month and the second month in patients from the PFD + M-DDO group was observed. Specifically, molecules involved in the synthesis, formation, and organization of granulation tissue or extracellular matrix were overincreased.

COL-1*α* increased its expression 50-fold at month 1 (*p* = 0.020) and 69-fold at month 2 (*p* = 0.050) compared with baseline values and showed a significant difference with the KTS group in both months (*p* = 0.030 and *p* = 0.018, resp.) (Table 3) Additional genes of this nature were COL-4, TGF-*β*1, and TGF-*β*3.

COL-4 had a significant increment with PFD + M-DDO in the first month (*p* = 0.001), which had a statistical difference versus KTS (*p* = 0.020). TGF-*β*1 had a significant improvement in the second month (*p* = 0.036) in the PFD + M-DDO group with respect to baseline value, presenting a statistical difference versus the KTS group (*p* = 0.018). TGF-*β*3 had an increase of 15.6-fold in the second month (*p* = 0.013) in the PFD + M-DDO group and showed a significant difference against the KTS group (*p* = 0.022) (Table 3); this is important since it has been shown that it is a key molecule in tissue regeneration.

Remarkably, PFD + M-DDO patients showed a 110-fold increase in ACTA2 gene expression (*p* = 0.019), which codifies for alpha-smooth muscle actin (*α-*SMA), a contractile protein.

Noncollagenous proteins such as elastin and fibronectin, needed to structure a granulation tissue scaffold, were found upregulated over baseline values as well.

KGF was also overincreased in PFD + M-DDO treatment at months 1 (*p* = 0.040) and 2 (*p* = 0.007) and presented a significant difference with KTS (*p* = 0.031) in month 2.

Finally, induction of angiogenesis needed for the formation of new blood vessels was represented by the expression of HIF-1*α*, HIF-1*β*, and VEGF.

HIF-1*α* showed a significant increase in the PFD + M-DDO group in month 1 (*p* = 0.007), with a statistical difference from the KTS group (*p* = 0.041); although no differences were found in the expression of HIF-1*β* and VEGF between groups, a clear tendency for increased VEGF was noted.

Gene expression in the two groups compared with baseline correlates with ulcer resolution. However, it was clear from our observations that patients treated with PFD + M-DDO expressed those molecules in a faster and stronger fashion, which is consistent with the fact that 43.8% of ulcers in PFD + M-DDO patients fully healed at three months.

### 5.4. Alpha-SMA Immunohistochemistry

**Figure 4:**
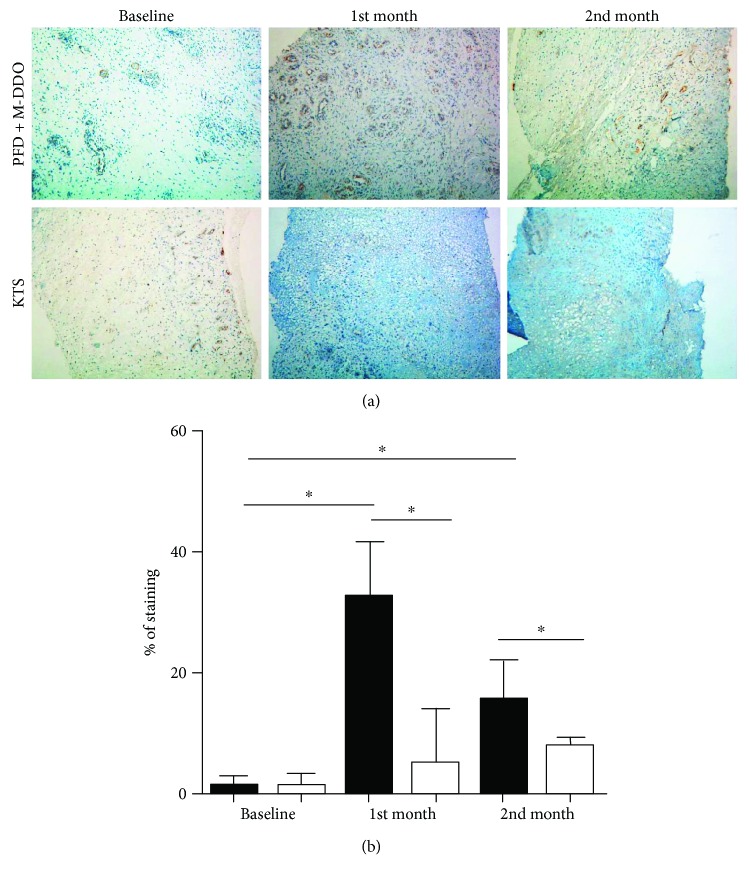
*α*-SMA immunostaining. Immunohistochemistry for a-SMA was performed on ulcer biopsies. (a) Representative microphotographs of diabetic foot ulcer tissue showed reactivity for anti-human a-SMA antibody. Magnification 20x. (b) Percentage of staining in ulcers by *α*-SMA (mean ± SD). *p* ≤ 0.05. Statistical significance is achieved between baseline values for PFD + M-DOO when compared to the first and second months of treatment. *p* ≤ 0.05. Also, a statistical difference between treatments is seen at the first and second months after therapy. Asterisk (∗) shows the significant difference between the bars shown.

Figure 4 shows the protein expression of *α*-SMA, a highly contractile protein. PFD + M-DDO-treated patients showed a substantial increase of this protein as compared with patients administered with KTS.

### 5.5. Safety and Tolerability

No serious adverse events were detected in the PFD + M-DDO group. 23.7% of the patients reported tolerable pain in the ulcer that was mentioned during the course of treatment, but it was not related to the medication.

In the KTS group, 28.2% of the patients reported tolerable pain. Of these, only one patient presented pain probably because of the drug administered. Intolerable pain was detected in one of the patients immediately after receiving the drug, which caused his elimination. The aforesaid pain subsided after ceasing application.

All patients included in this clinical trial had similar demographics and biochemical profiles that were not modified after receiving the treatments.

## 6. Discussion

KTS has been tried for diabetic foot ulcer treatment with proven effect on decreasing ulcer area between 59 and 60% [27–29], though the effects on inflammation and risk of infection have not been completely determined.

In this same context, due to the anti-inflammatory and antioxidant role and its dynamic effect on matrix extracellular synthesis, pirfenidone is a potential drug for diabetic foot ulcer treatment.

Our results in this double-blind, randomized, active-controlled protocol are consistent with the abovementioned Janka-Zires et al. report. They used pirfenidone as a pharmacological treatment of noninfected chronic diabetic ulcer for 16 weeks and reported a 93% reduction of ulcer size when compared to control with conventional treatment of only 21.8% [17]. Our findings showed a 100% reduction of median RUV in PFD + M-DDO group at 16 weeks, and PFD + M-DDO proved to be more effective than ketanserin in noninfected ulcers since RUV was reduced by 99.7% with the use of PFD + M-DDO, whereas with the use of KTS, it was only 60% in the third month.

These clinical results correlated with the wound healing histopathological score where PFD + M-DDO dramatically reduced inflammatory infiltrate, which is consistent with previous findings in different tissues [35]. Furthermore, in this specific clinical setting of diabetic ulcer, PFD + M-DDO unexpectedly increased collagen deposition. Abundant mature collagen, which looked organized and composed of aligned fiber bundles, was observed in the course of the trial. Thus, granulation tissue recruitment took place faster [36].

It is known that neuropathy, peripheral vascular disease with ischemia, coupled with hyperglycemia and infection are associated with a failure to heal and possible amputation [37]. All these complications are associated with chronic sensor-motor neuropathy and vascular disease [6], TGF-*β*1 and HIF-1*α* downregulation [7, 8], high levels of TNF-*α* and oxidative stress [10], delayed expression of KGF [11], and impaired immune function.

In this context, a major amount of healed ulcers from the second month up to six months of treatment in the PFD + M-DDO group could be influenced by the guided gene expression of important genes.

The increased expression of KGF in PFD + M-DDO treatment could be related to faster reduction of RUV since this powerful mitogen of epithelial cells is very important in wound healing [38]. TGF-*β*3 and TGF-*β*1 in turn promote fibroblast migration and granulation tissue/extracellular matrix maturation [36]. PFD + M-DDO did improve expression of COL-1*α*, which eventually will form collagen bundles where fibroblasts will migrate in the proliferation phase [39]. *α*-SMA is an indicator of the transformation of fibroblast to myofibroblast since it promotes cell contractility, which would help to close the wound and maintain its structure [40]. The expression of *α*-SMA in our patients treated with PFD clearly correlates with the expression of its cognate mRNA and further sheds light on the notion that this molecule is acting to “reinforce” the resilience of wound closure.

On the other hand, it is critical to consider using an antiseptic agent together with a wound healing “regenerator,” given that infections are a major cause of the occurrence of amputations in diabetic patients [41], considering that 77% of patients submitted to amputation will die in the first five years [5]. It is considered that M-DDO increased the beneficial effect of pirfenidone since M-DDO is a potent germicide, which prevented wound-opening recurrence observed in the ketanserin group characterized by infection and others causes. Meanwhile, no patients in the PFD + M-DDO group neither went back to ulcer evolution nor presented infection nor necrosis. According to these results, the combination of both drugs (PFD + M-DDO) presents an advantage over ketanserin, especially in preventing infections and accelerating and improving ulcer resolution.

The insights gained from this type of assessment are expected to facilitate the development of novel therapies by stratifying their specific contributions to the wound healing process in time and in a stage-specific manner.

## Figures and Tables

**Table 1:** Patient baseline demographic and clinical characteristics.

Groups characteristic	Baseline		*p* value
	PFD + MDD-O	KTS	
Age (years)	57.3 ± 12.6	51.7 ± 9.9	0.069
Gender *n* (%)	Male 18 (76.9%)	Male 12 (57.7%)	0.139
	Female 5 (23.1%)	Female 18 (42.3%)	
Years with diabetes	14 ± 9.1	17 ± 10.4	0.416
Months with ulcers	8.6 ± 2.7	6.5 ± 2.2	0.989
Smoking	7 (26.9%)	2 (7.7%)	0.140
Glucose (mg/dL)	139.1 ± 71.7	152.8 ± 85.7	0.597
HDL (mg/dL)	31.4 ± 14.7	38.9 ± 11.8	0.840
VLDL (mg/dL)	27.5 ± 11.6	42.6 ± 40.3	0.211
LDL (mg/dL)	78.9 ± 13.5	101.7 ± 39.1	0.107
Creatinine clearance/24 h (mL/min)	59.9 ± 20.9	74.2 ± 40.8	0.517
Urea (mg/dL)	37.5 ± 24.4	46.0 ± 21.4	0.173
Creatinine (mg/dL)	1.0 ± 0.5	1.6 ± 1.1	0.128
ALT (U/L)	14.4 ± 5.4	25.2 ± 15.4	0.073
AST (U/L)	16.0 ± 3.2	23.4 ± 15.0	0.404
GGT (U/L)	37.1 ± 12.9	90.4 ± 14.5	0.973
VSG (mm/h)	78.1 ± 39.2	64.2 ± 29.2	0.456

The table shows the baseline demographic characteristics of the participants. The groups were homogeneous and comparable at the beginning and end of the trial.

**Table 2:** Wound healing histopathological score.

Treatment	Baseline	Month 1	Month 2
PFD+M-DDO	11 (9.5–15)	12 (10–15)	**15.5 (14.3–16)** ^**+**^^∗^
KTS	11 (10–13)	11 (10–12.5)	**12.5 (11–16.3)** ^∗^
*p* between treatments	*p* = 0.621	*p* = 0.273	*p* = 0.050

The table shows the wound healing histopathological score of each treatment. An increase in the score was observed in the second month for the PFD + M-DDO group, which is statistically significant between the groups. Data are presented in median (interquartile range). ^+^Significant difference with baseline. ^∗^Significant difference between groups.

**Table 3:** Gene Expression.

Gene	Baseline	PFD + M-DDO		KTS	
		1st month	2nd month	1st month	2nd month
Col-1*α*	1	**49.00** ± **38.06** ^**+**^^∗^	**69.15** ± **62.02** ^**+**^^∗^	**1.08** ± **0.48** ^∗^	**0.17** ± **0.08** ^∗^
Col-4	1	**22.09** ± **9.02** ^**+**^^∗^	2.35 ± 0.52	**13.59** ± **5.70** ^∗^	1.02 ± 0.62
TGF-*β*1	1	1.23 ± 0.37	**2.99** ± **1.64** ^**+**^^∗^	1.97 ± 0.56	**0.80** ± **0.26** ^∗^
TGF-*β*3	1	6.66 ± 4.83	**16.62** ± **15.02** ^**+**^^∗^	1.50 ± 0.40	**0.73** ± **0.33** ^∗^
*α*-SMA	1	**111.41** ± **32.44** ^**+**^	14.53 ± 8.81	**2.74** ± **1.34** ^**+**^	3.02 ± 2.77
Elastin	1	356.53 ± 212.17	122.37 ± 77.39	8.29 ± 2.74	13.02 ± 6.09
Fibronectin	1	6.26 ± 2.25	4.39 ± 2.34	0.79 ± 0.23	1.44 ± 0.25
MMP-1	1	2.23 ± 0.95	11.25 ± 9.11	3.38 ± 1.56	1.49 ± 0.72
KGF	1	**8.75** ± **5.20** ^**+**^	**5.42** ± **3.45** ^**+**^^∗^	11.02 ± 5.81	**21.59** ± **16.97** ^∗^
HIF-1*α*	1	**1.59** ± **0.51** ^**+**^^∗^	1.95 ± 0.82	**1.51** ± **0.31** ^∗^	1.09 ± 0.49
HIF-1*β*	1	1.55 ± 0.49	1.32 ± 0.52	2.95 ± 1.33	1.09 ± 0.29
VEGF	1	1.27 ± 0.37	2.07 ± 1.11	2.27 ± 0.60	0.92 ± 0.42

The table shows gene expression at baseline, first, and second months of treatment. Gene expression was normalized against the housekeeping gene 18S. Relative quantification was performed using the 2^−ΔΔCT^ method. Data are expressed as mean + SEM. ^+^Significant difference with baseline. ^∗^Significant difference between groups.
